# Subcortical responses to music and speech are alike while cortical responses diverge

**DOI:** 10.1038/s41598-023-50438-0

**Published:** 2024-01-08

**Authors:** Tong Shan, Madeline S. Cappelloni, Ross K. Maddox

**Affiliations:** 1 Department of Biomedical Engineering, University of Rochester, Rochester, NY USA; 2 Del Monte Institute for Neuroscience, University of Rochester, Rochester, NY USA; 3 Center for Visual Science, University of Rochester, Rochester, NY USA; 4 Department of Neuroscience, University of Rochester, Rochester, NY USA

**Keywords:** Cortex, Midbrain, Auditory system

## Abstract

Music and speech are encountered daily and are unique to human beings. Both are transformed by the auditory pathway from an initial acoustical encoding to higher level cognition. Studies of cortex have revealed distinct brain responses to music and speech, but differences may emerge in the cortex or may be inherited from different subcortical encoding. In the first part of this study, we derived the human auditory brainstem response (ABR), a measure of subcortical encoding, to recorded music and speech using two analysis methods. The first method, described previously and acoustically based, yielded very different ABRs between the two sound classes. The second method, however, developed here and based on a physiological model of the auditory periphery, gave highly correlated responses to music and speech. We determined the superiority of the second method through several metrics, suggesting there is no appreciable impact of stimulus class (i.e., music vs speech) on the way stimulus acoustics are encoded subcortically. In this study’s second part, we considered the cortex. Our new analysis method resulted in cortical music and speech responses becoming more similar but with remaining differences. The subcortical and cortical results taken together suggest that there is evidence for stimulus-class dependent processing of music and speech at the cortical but not subcortical level.

## Introduction

Music and speech are two uniquely human classes of sounds. Recent studies have reported that the human brain has specialized responses to music and speech versus other sound stimuli^1–4^. These sounds, once they reach our ears, spark a cascade of neural activity beginning with basic encoding of acoustics and eventually activating high level brain functions, such as understanding, memory, and emotion^5^. However, how this transformation from encoding to perception to cognition happens along the auditory pathway, and how the process differs at each stage between music and speech, remains unclear.

Previous studies investigating the human brain processing of music and speech have mostly focused on the cortex. While neural overlap of music and speech processing has been suggested, several studies have noted differences^6–10^. Music and speech stimuli have different spectral and temporal modulations, and this has been reported to underlie asymmetrical processing^11,12^. A study reconstructing the envelope from electroencephalogram (EEG) responses found different cortical envelope tracking between speech and music^13^. Studies using fMRI have also revealed selectivity patterns of neural populations from auditory cortex in response to music and speech^2,4^. Other studies, however, have investigated the processing of syntax and structure in speech and music and found shared networks, suggesting that their syntactic integration may share similar mechanisms^14–18^.

While observable at cortex, it is still unknown at which stage of auditory pathway the differences in encoding between music and speech processing first arise. At the subcortical level, there is comparatively limited work comparing music and speech responses. Some studies have examined low-level encoding of short speech and music sounds by analyzing the transient onset response and the frequency-following response (FFR)^19–24^. These studies have revealed some relationship between the subcortical response and the spectral or temporal attributes of music and speech. However, the purely subcortical origin of the FFR is debatable, and it is likely a mixture of cortical and subcortical generators^25^. Further, all of these studies were restricted to short stimuli such as a single vowel or phoneme for speech, or a single pitch (or pitch interval/chord) for music, none of which represent the richness of natural music or speech.

The auditory brainstem response (ABR) can be used to characterize subcortical activity^26^. It comprises the first ~ 10 ms of the auditory evoked potential (whose later waves are cortical in origin), and its component waves can be attributed to distinct stages of the auditory pathway according to their latency^27^. Wave I, for example, corresponds to the activity of auditory nerve, wave III to cochlear nucleus, and wave V to later areas such as inferior colliculus and lateral lemniscus^27^. While its traditional measurement requires many repetitions of short stimuli such as clicks or tone bursts, several techniques have recently been developed that allow the derivation of the ABR to continuous and non-repetitive stimuli, with some caveats. Some methods have been developed for speech but do not generalize to polyphonic music^28–30^. Another study used the subcortical temporal response function (TRF) to measure responses to each line in two-part melodic musical pieces, but was not tested with other music^31^. A third, similar technique takes the half-wave rectified audio waveform as the input to a linear system and computes the evoked potential^32^. In that final study, the derived response to speech showed a clear wave V with a high degree of similarity in morphology and latency to the click-evoked ABR, but with earlier waves (wave I–IV) “smeared” together. Despite this drawback, this paradigm has the advantage of making no assumptions about the input stimulus (e.g., that it is speech, or that it has a definable fundamental frequency), and crucially for the present study allows both subcortical and cortical responses to be estimated from the same EEG recording.

**Figure 1:**
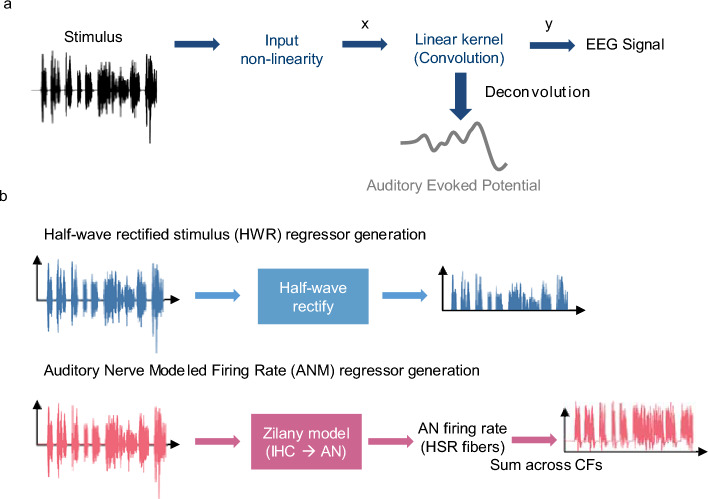
The encoding model and the regressors that are used in deconvolution. (**a**) The encoding model. The stimulus is first processed with a nonlinear function and then the processed stimulus is used as the input $x$ (i.e., regressor). The EEG data is considered as the output $y$. The ABR or AEP is the impulse response from the linear kernel and can be derived by deconvolving the EEG data and the regressor. (**b**) Regressor generation process. Upper, the half-wave rectified stimulus (HWR). Lower, the Auditory Nerve Modeled firing rate (ANM). IHC = inner hair cell, AN = auditory nerve, HSR = high spontaneous rate, CF = characteristic frequency.

The deconvolution technique we used in this paper is based on Maddox and Lee^32^, where we defined an encoding model of the auditory evoked potential as shown in Fig. 1. The stimulus with a non-linearity applied (i.e., regressor) was the input $x$, the EEG signal was the output $y$, and the ABR was the impulse response of a linear system which transforms $x$ into $y$.

In this study we first used an existing method that uses the half-wave rectified stimulus (HWR) as the regressor for deconvolution, but found it worked poorly for musical stimuli. This led us to develop a new analysis method which we determined quantitatively to be superior. We were then able to derive and compare music and speech responses at the subcortical and cortical levels, shedding light on where higher order attributes such as stimulus class likely first impact encoding of acoustics.

## Results

We collected EEG data from 22 adults with normal hearing thresholds who gave informed consent. We used six different genres of music (including classical, jazz, acoustic, hip-hop, metal, and pop) and six types of speech (Chinese audiobook, English audiobook, interview, instructional lecture, news, and presentation) as stimuli. See Materials and Methods for details.

In the following subsections we first describe the conflicting findings from the old and new subcortical deconvolution methods. We then reconcile that conflict by determining which metric is more accurate. Finally, we compare the cortical responses to speech and music.

### Old method: subcortical music and speech responses are uncorrelated

We first obtained the music- and speech-evoked ABRs using deconvolution with the half-wave rectified stimulus as the regressor (HWR; Fig. 1b upper) as in Maddox and Lee^32^, which was able to derive an ABR with wave V for continuous speech. We note that despite comparing two methods here, this older method is the only one we had at our disposal at the beginning of the study, and the results motivated the development of the new method described later.

**Figure 2:**
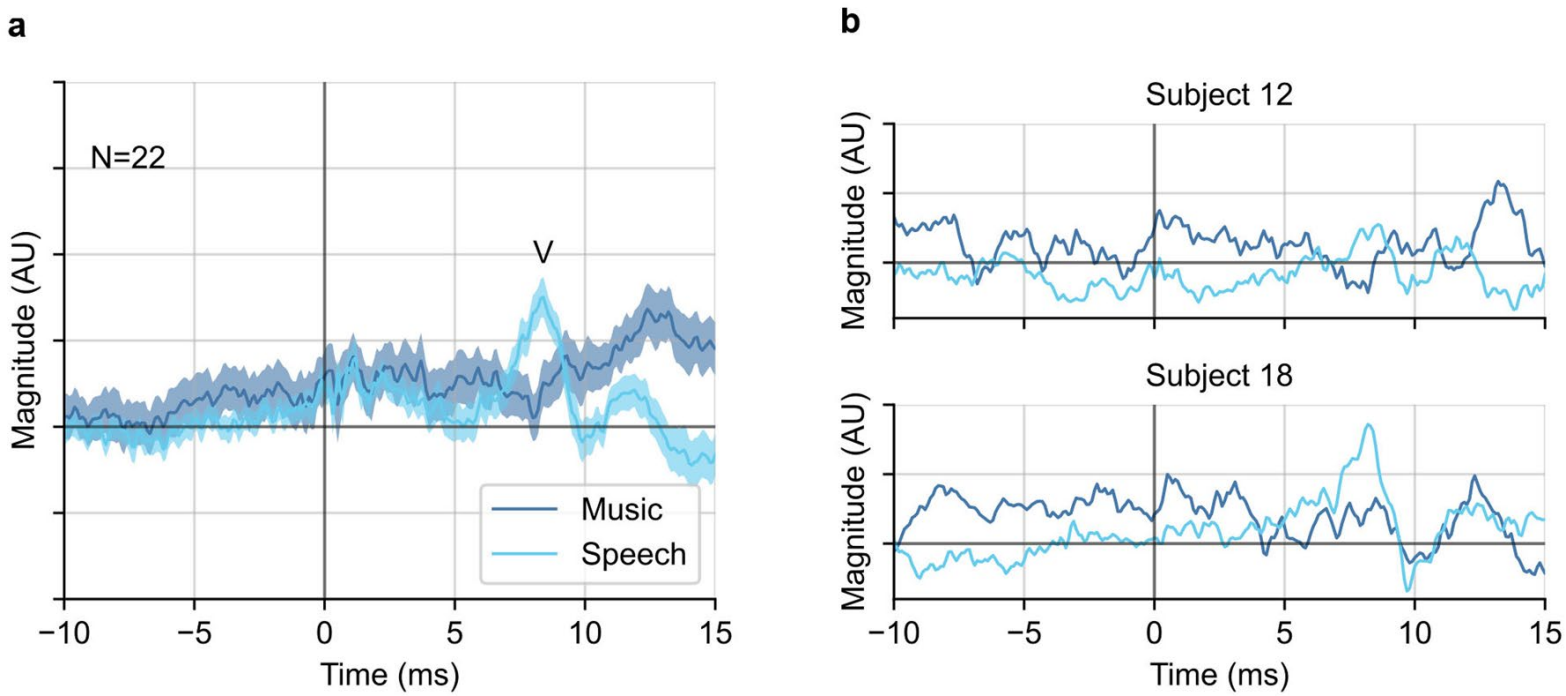
General music- and speech-evoked ABR waveforms using the half-wave rectified stimulus as the regressor in deconvolution. (**a**) The grand averaged general music- and speech-evoked ABR waveforms. Wave V in the speech response at 7.5 ms is annotated. The plotted waveforms were low-pass filtered at 1500 Hz. Shaded areas show ± 1 SEM (n = 22). (**b**) Two example individual subject responses.

The general music-evoked ABRs for each subject were calculated by averaging the responses to all six genres of music stimuli. The same process was done with the speech-evoked ABRs. Figure 2a shows the grand average waveforms of the general music- and speech-evoked ABRs in a time range from − 10 to 15 ms. Wave V is present in the speech-evoked ABR with a peak at around 7.5 ms latency. However, wave V was absent from the music-evoked ABR. An extended time range from − 50 to 300 ms of the response can be seen in Supplementary Figure S2a.

Observing individual responses, it is clear that there is substantial noise. Figure 2b shows the examples of two individuals’ responses (subject 12 and subject 18). Subject 18 showed a distinct wave V in the speech response but not in music. Subject 12 had a weaker wave V in the speech response only slightly larger than the noise, and a music response that was again missing wave V. In our analysis, the median signal-to-noise ratio (SNR) in time range [0, 15] ms for speech responses was 0.05 dB, but over half subjects failed to show a measurable SNR for the music response. All individual subject responses are shown in Supplementary Figure S1.

We then computed the correlation between each subject’s music and speech responses to assess their similarity^30^. The waveforms for music- and speech-evoked ABR were essentially unrelated, with a median (interquartile range) correlation coefficient across subjects of 0.00 (− 0.13 to 0.05).

We considered two potential reasons for the stark difference between the music- and speech-evoked ABR: (1) The brain is in different states when listening to music and speech, leading to different encoding of the two sounds’ acoustics. (2) Music and speech have different acoustics^33,34^ which need to be fully accounted for in deconvolution. The poor waveforms (i.e., low SNR and weak or absent wave V) of music responses may be due to the acoustical features inadequately captured by HWR.

### New method: subcortical music and speech responses are highly correlated

To address the possibility of inadequate analysis leading to the differences reported above, we used the auditory nerve modeled firing rate (ANM; Fig. 1b, lower) generated from the Zilany et al. model^35,36^ as a second regressor to derive the ABR. This computational model is a phenomenological model of the early auditory pathway, which transforms a stimulus waveform to a detailed human neural representation of that acoustical signal. This model incorporates the nonlinearities observed at each stage of the auditory periphery, including nonlinear tuning, compression, suppression, level-dependent phase, rate saturation, adaptation, synchrony capture, etc.^35,36^ (see Materials and Methods). We used the ANM regressor in hopes that it would account for the potential interaction of auditory peripheral nonlinearities with the differing overall acoustics between music and speech.

**Figure 3:**
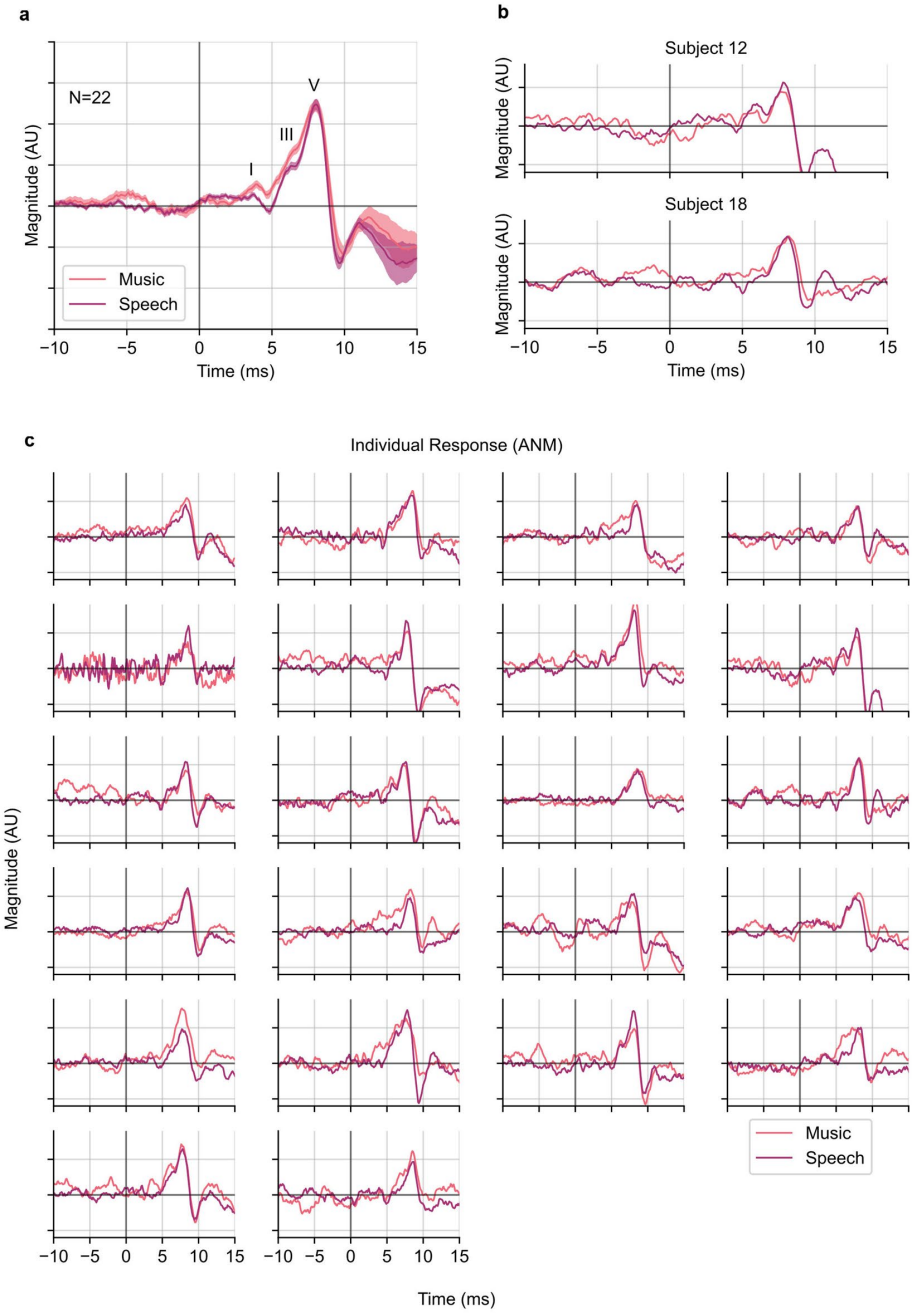
General music- and speech-evoked ABR waveforms using the ANM regressor. (**a**) The grand averaged general music- and speech-evoked ABR waveforms. Wave I, III, and V are annotated. The plotted waveforms were low-pass filtered at 1500 Hz. The shading areas show ± 1 SEM (n = 22). (**b**) Two example individual subject responses, as in Fig. 2. (**c**) Responses of all subjects using the ANM regressor. All subjects showed clear wave V and some also showed clear wave I and wave III.

Figure 3a shows the grand average waveforms of the general music- and speech-evoked ABRs. Unlike with the HWR regressor, waves I, III, and V of the canonical ABR waveform were all identifiable for both the music- and speech-evoked ABR. Additionally, all individuals’ responses showed clear wave V, with many also showing earlier waves (Fig. 3c). The average waveforms represent a marked improvement in morphology over the HWR regressors. All subjects’ responses showed good SNRs, with a median SNR of 9.1 dB for music responses and 12.7 dB for speech. Also notable is that subject 12 (Fig. 3b, upper), who previously had a poor speech response and no music response showed strong a wave V in both responses, and also a visible wave I in the speech response. An extended time range from − 50 to 300 ms of the ANM response can be found in Supplementary Figure S2b.

**Figure 4:**
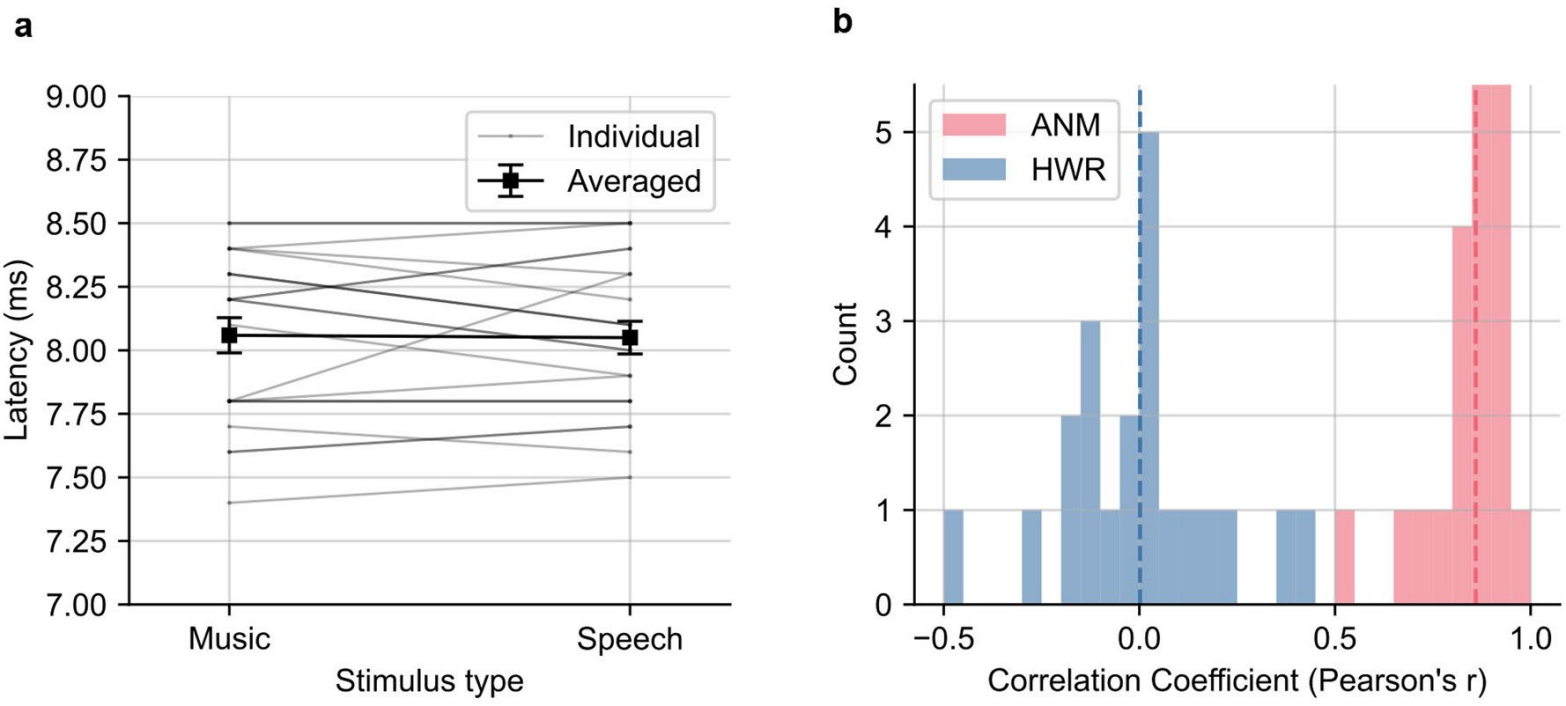
Comparison between the music- and speech-evoked ABRs. (**a**) Latency of Wave V in music- and speech-evoked ABRs derived from the ANM regressor. The grey lines are individual subject latencies. Darker lines indicate subjects with same latencies and overlapping with each other. The black line shows the average latencies, error bars are ± 1 SEM. (**b**) Histogram of music-speech Pearson correlation coefficients of ABRs derived from the HWR and ANM. The two dashed lines are the median coefficients of the two distributions. The median (interquartile range) for HWR and ANM responses are 0.00 (− 0.13 to 0.05) and 0.86 (0.84–0.90). The distribution of correlations between music- and speech-evoked ABR for the ANM regressor is significantly higher than the HWR (*p* = 2.4 $\times$ 10^−7^; one-tailed Wilcoxon signed-rank test).

To gauge the similarity of the waveforms across stimulus types, we first measured the wave V latency from the ABRs (Fig. 4a). The mean ± SEM latency of wave V of the music-evoked ABR was 8.1 ± 0.1 ms, very close to that of the speech-evoked ABR, which was 8.0 ± 0.1 ms (*p* = 0.82; two-tailed paired t-test). This analysis could not be completed for the HWR regressor because there was generally no wave V for the music-evoked ABR.

We next computed the correlation between the music and speech response waveforms, as above for the HWR regressor. The median (interquartile range) of the Pearson correlation coefficients between the average music-evoked ABR and average speech-evoked ABR waveform was 0.86 (0.84–0.90). These correlations indicate a high degree of similarity, which was significantly higher than that of the responses derived from the HWR regressor as shown in Fig. 4b (*p* = 2.4 $\times$ 10^−7^; one-tailed Wilcoxon signed-rank test). When examining the responses to each genre of music and speech, there were also no major response differences in morphology within the two stimulus categories, though there was more variation across genre for music responses—a statement which is also true of the stimuli themselves (see the pair-wise correlation in Supplementary Figure S3).

To get a sense of the level of similarity between the music and speech responses, we can compute a split-set correlation, where we build randomized split sets of responses that comprise music and speech responses in equal amounts. We can then compute the correlation between them, with any unexplained variance attributable purely to noise. When we did so, we obtained a median (interquartile range) correlation of 0.90 (0.85–0.94). This is slightly higher than the music-speech correlation of 0.86 (*p* = 0.0026; Wilcoxon sign-rank test), meaning that 91% of the explainable variance (noise-adjusted *r*^2^ = 0.86^2^/0.90^2^ = 0.91; noise-adjusted *r* = 0.95) between music and speech responses is accounted for, but the remaining 9% is due to something other than randomness.

### Which method to trust? ANM regressor yields better predictions of subcortical activity than HWR

At this point in our study, we were faced with two conflicting conclusions from different analyses of the same data. Using the ANM regressor we would conclude that there is very little difference between the subcortical response to music versus speech. Using the HWR regressor, however, we would conclude that the subcortical response to music is entirely different from that to speech. Both of these responses are models of the EEG response—we thus will decide which result better reflects the underlying processing of speech and music based on the predictive power of each model, assessed in two ways.

**Figure 5:**
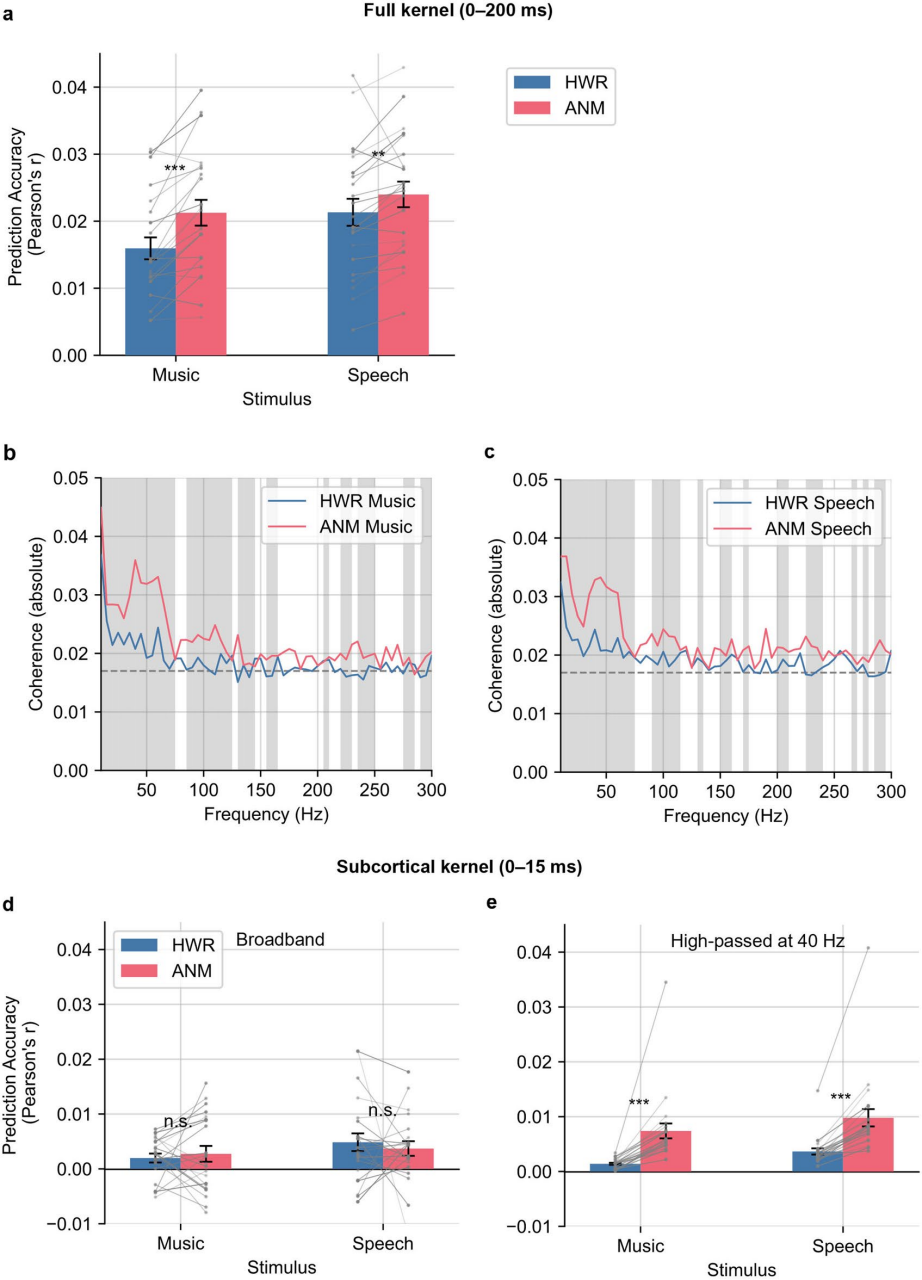
Prediction accuracies for the HWR and ANM regressor. (**a**) Broadband correlation coefficient with full kernel (0–200 ms). The bars are averaged accuracy across subjects with error bars showing ± 1 SEM. The prediction accuracy of the ANM regressor is significantly higher than that of HWR (music *p* = 9.8 × 10^−6^, speech *p* = 0.0060; one-tailed paired t-test). (**b**) Spectral coherence for music trials with full kernel. (**c**) Spectral coherence for speech trials with full kernel. The solid lines are the median absolute value of spectral coherence for each regressor in each frequency bin. The shaded areas show frequencies where the coherence for ANM regressor is significantly higher than the HWR regressor (*p* < 0.05; Wilcoxon signed-rank test, FDR corrected). The dashed lines indicate the noise floor (see Materials and Methods for details). (**d**) Broadband correlation coefficient with subcortical kernel (0–15 ms). There is no significant difference in the prediction accuracy between HWR and ANM regressor (*p* > 0.05; two tailed paired t-test). (**e**) Correlation coefficient of high-pass filtered EEG with subcortical kernel. The prediction accuracy of the ANM regressor is significantly higher than that of HWR (music *p* = 6.8 × 10^−5^, speech *p* = 7.1 × 10^−6^; one-tailed paired t-test).

We performed a Pearson correlation to compare the accuracy of the predicted EEG signal to the recorded one for each regressor, as is commonly done in cortical TRF studies^37,38^. We first used the derived waveforms from time range [0, 200] ms as a full kernel, and convolved it with the corresponding regressors to get the predicted EEG data. The averaged prediction accuracies of the HWR and ANM regressors are shown in Fig. 5a. The HWR regressor showed correlations of 0.016 and 0.021 for music and speech, respectively. The ANM regressor showed higher correlations of 0.022 and 0.023. For both music and speech, the ANM prediction was better than the HWR prediction (*p* = 9.8 × 10^−6^ and *p* = 0.0060, respectively; one-tailed paired t-test). We did not test our predictions out of sample, but both models had the same amount of training data and the same number of parameters, so the comparison of correlation coefficients is still indicative of relative model quality.

The correlation between predicted and recorded EEG is a gross, broadband measure and will reflect both subcortical and cortical activity. To account for this, we then used the derived waveform from the response time range of [0, 15] ms as the subcortical kernel, aiming to isolate and examine the purer subcortical contribution. When comparing the performance of the ANM to the broadband correlation (Fig. 5d), there was no visible advantage. However, as we high-passed the signals at 40 Hz, we observed a marked improvement in the ANM’s prediction in comparison to HWR (music: *p* = 6.8 × 10^−5^, speech: *p* = 7.1 × 10^−6^; one-tailed paired t-test; Fig. 5e).

The response to sounds represents only a very small portion of recorded EEG activity, which leads to generally low correlation coefficients. To put our correlation values in context, we used data from a different experiment to determine what the best-case correlation values might be. We analyzed responses from 61 subjects to repeated /da/ and /mi3/ syllables^39^. We computed the average response to each stimulus for each subject and modeled the EEG response by placing this averaged response in a sequence aligned with the stimulus start times. The median predictive correlation was 0.037 for both stimuli. It is thus very unlikely that an experiment such as ours that fits a stimulus-agnostic general forward model could do any better than that. Even a perfect model of the auditory response would yield very low correlation values when used to predict samples of EEG recordings due to their endemic noise.

We also performed spectral coherence analysis, which provides a normalized similarity between the predicted EEG and the real EEG data on a per-frequency basis (see Materials and Methods for details). Figure 5b and c show the absolute value of the coherence using the full kernel of frequency range of [0, 300] Hz for music and speech trials, respectively. The ANM regressor surpasses the HWR regressor for much of the frequency range. With the subcortical kernel, the coherence improvement of the ANM regressor remains, especially in the range of [40, 60] Hz and [90, 110] Hz for both speech and music trials (Supplementary Figure S4). Coherence at these frequencies is likely driven by the faster, earlier parts of the auditory system, especially considering the jump in subcortical correlation coefficients when high-passing at 40 Hz (Fig. 5e).

Overall, we found highly similar subcortical responses for music and speech using the ANM regressor in deconvolution. When compared to the HWR regressor, the correlation between the responses to the two types of stimuli was significantly higher. These findings suggest that when accounting for detailed peripheral nonlinearities, subcortical encoding of music and speech acoustics is strikingly alike (noise-adjusted *r* = 0.95).

### New method increases similarity between cortical music and speech responses but differences remain

**Figure 6:**
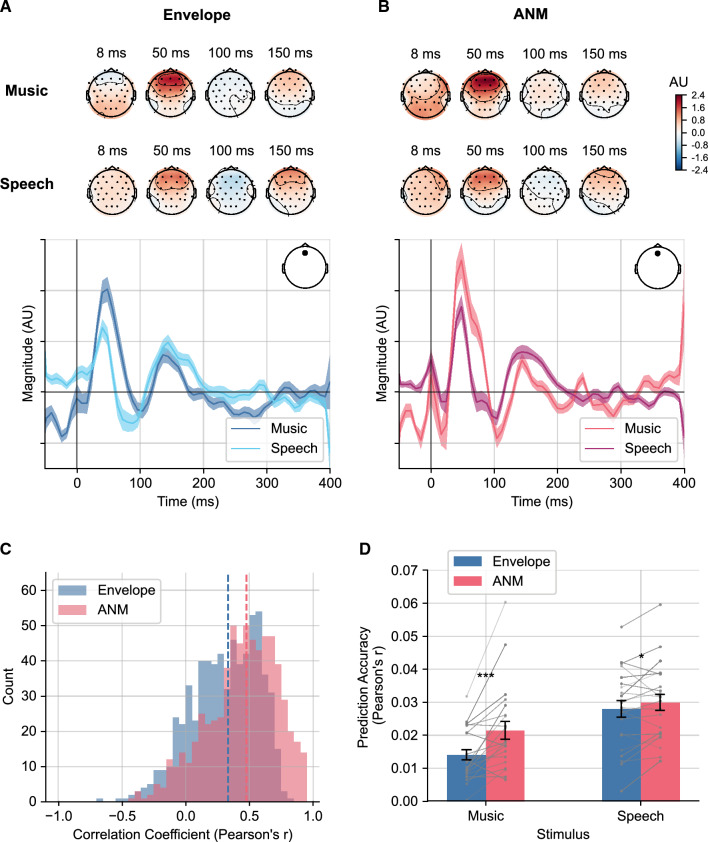
TRF derived from music and speech stimuli from cortical activity. (**a**) TRFs derived from envelope regressor. (**b**) TRFs derived from ANM regressor. Topographies are shown at time lags 8, 50, 100 and 150 ms. The TRF weights for music and speech are shown for Fz channel in blue and pink lines for envelope and ANM regressor, respectively. (**c**) Correlation coefficient between TRFs from music and speech derived from envelope and ANM regressor. The histogram shows the distribution of pooled correlation coefficients of each EEG channel and each subject. The dashed lines are the median coefficients for the two regressors. The median (interquartile range) for envelope and ANM is 0.33 (0.13–0.52) and 0.48 (0.29–0.65), respectively. (**d**) Prediction accuracy for the envelope and ANM regressor. The bars are averaged accuracy across subjects. Dots with lines are individual subject coefficients. The correlation coefficient (mean ± SEM) for envelope is 0.014 ± 0.002 for music and 0.028 ± 0.003 for speech; The correlation coefficient for the ANM regressor is 0.021 ± 0.003 for music and 0.030 ± 0.002 for speech. The prediction accuracy of the ANM regressor is significantly higher than that of envelope (music *p* = 3.4 × 10^−4^, speech *p* = 0.039; one-tailed paired t-test).

We next assessed the cortical responses to the same music and speech from data acquired during the same recording session. We reasoned that by better representing peripheral encoding the ANM regressor would provide better cortical responses as well. In addition to EEG recorded from the standard ABR montage, we also collected 32-channel scalp EEG data suitable for assessing cortical responses. These data were processed as described by Crosse et al.^40^. We utilized a linear modeling framework—the temporal response function (TRF)—which has been used to derive the cortical response to natural sounds in previous studies^37,41^. The TRFs were determined by fitting a ridge regression model of the recorded EEG using either the ANM regressor or the acoustic envelope (as done in many previous studies^37,42–44^) as the input, using leave-one-out cross-validation to determine the regularization coefficient (see Materials and Methods for details). Figure 6a and b show the fitted TRF topography from various peak time points and the weights from a selected channel derived from music and speech stimuli using either the envelope or ANM regressor.

There is a higher degree of similarity between music and speech encoding at the cortical level when using the ANM regressor versus the envelope, although the change is less pronounced compared to the ABRs. At the cortical level, the change in regressor results in a change in the median (interquartile range) music-speech response correlation from 0.33 (0.13–0.52) to 0.48 (0.29–0.65). The distribution of correlation coefficients for each of the two regressors is shown in Fig. 6c. Additionally, we applied a linear mixed effect model with the correlation coefficients serving as the dependent variable, regressor type as the fixed effect, and subjects and EEG channels as random effects (see Materials and Methods for details). The model revealed a main effect of regressor type (*p* < 2 $\times$ 10^−16^), confirming the difference.

The prediction accuracies (correlation coefficient) from the two regressors are shown in Fig. 6d. Our findings indicate that the ANM exhibited significantly higher accuracy in predicting music trials (*p* = 3.4 × 10^−16^). It showed slightly but significantly better accuracy in predicting speech as well (*p* = 0.039; one-tailed paired t-test). Notably, just as with the subcortical analysis (though to a lesser degree), the ANM regressor provided better accuracy and suggests more similar responses to speech and music than the basic acoustical regressor.

**Figure 7:**
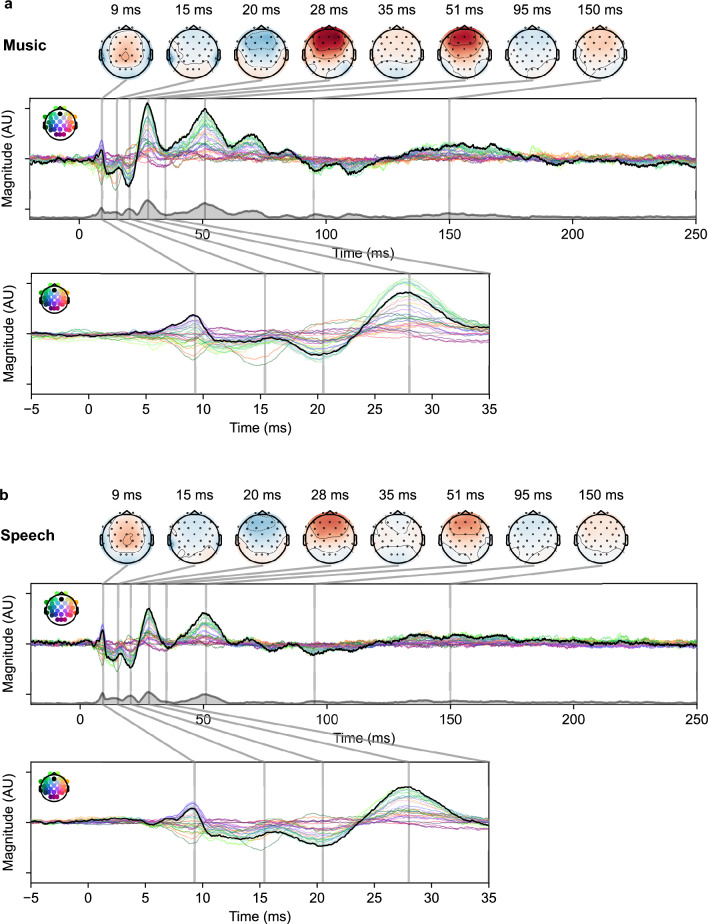
The cortical response derived through deconvolution from the ANM regressor. (**a**) Music-evoked cortical responses. (**b**) Speech-evoked cortical responses. Topographies are selected from the time points of important peaks. The black lines are shown for Fz channel with other channels shown in colors. The thick gray line with light gray under-line area represents the global field power (GFP) of the responses. The early responses in time window of [− 5, 35] ms are shown below in each panel.

An alternative approach to the regularized TRF paradigm is to directly derive the cortical responses through deconvolution in the same manner as for ABRs. As shown in Fig. 7, the general morphology of deconvolution response derived from ANM is similar to the TRF derived from the same regressor. The advantage of our deconvolution method is that, because no downsampling is needed, it allows for better visualization of early and middle latency components in addition to late cortical responses all as part of the same response waveform. These early and middle latency responses can reveal information about different stages of processing in the auditory pathway than the late response components^45,46^. While traditional TRFs could be computed to this detail using the ANM regressor, the high sampling rate would lead to extremely long computation times. In contrast, all that is needed to view cortical responses deconvolved from the ANM regressor is to extend the axis limits beyond the subcortical response to include longer latencies—no additional computation is needed.

In the TRF analysis, we also observed a higher degree of similarity between the cortical responses to music and speech when the auditory peripheral nonlinearity was accounted for (i.e., using the ANM regressor). It is also true that for TRF analysis, the ANM outperformed the traditionally used envelope as the regressor. These results indicate that the ANM regressor can help reduce the confounding of the cortical differences between the acoustical encoding of music and speech, although some discrepancies persist that may be due to differences in high-level cognitive processing or modulation of the encoding of acoustics^43,44,47^. Overall, the use of the ANM regressor represents a promising approach for cortical analysis, too.

## Discussion

Here we measured and compared brain responses to continuous music and speech in human listeners using deconvolution techniques. We first derived ABRs to a broad range of stimuli using the HWR regressor described previously by Maddox and Lee^32^. The resulting music- and speech-evoked ABRs from this regressor were markedly distinct, a result which led to two distinct hypotheses: (1) a difference in brain state when listening to the two stimulus classes that affects their subcortical encoding (as attention does for cortical encoding^48,49^, for example) or (2) suboptimal modeling of the subcortical response. We developed the ANM regressor to address this question and found that music and speech evoked nearly the same ABR, suggesting that the previously observed difference was due to issues with the HWR regressor, and not differential subcortical processing of music and speech. We next utilized this regressor in the derivation of cortical TRFs and found that while the ANM regressor accounts for some acoustical differences between music and speech responses, differences persist, implicating differentiated encoding between music and speech in the cortex where subcortical responses were highly alike.

It is important to note that deconvolution is by construction a linear process, and thus assumes the system being analyzed is linear. The auditory system, however, is replete with nonlinearities which are essential to its function. Therefore, when studying the auditory system with deconvolution, the choice of the “input” for the model (regressor) needs to be the stimulus processed with some nonlinear function that makes the model biologically meaningful. While HWR can be considered a nonlinearity that occurs in the cochlea, it is a very simple simulation and cannot fully capture the complicated nonlinearities that occur in the auditory periphery.

To overcome the limitations of the HWR regressor, we introduced the ANM regressor generated by a computational auditory periphery model^35,36^. This computational model comprehensively includes each early auditory stage from middle ear to cochlea to AN. Employing such a model allowed us to apply a more biologically plausible nonlinearity to each stimulus. As a result, we obtained highly similar (*r* = 0.86, noise-adjusted *r* = 0.95) music- and speech-evoked ABRs. The ANM regressor provided qualitatively better waveforms (i.e., higher SNR, easily discernable wave V), and comparing the prediction ability of the two regressors based on the broadband correlation and spectral coherence confirmed that the ANM regressor is indeed superior to HWR. In the case of the ABR, the best nonlinearity seems to be the one which most accurately models the early physiological nonlinearities. If there is indeed no specialized music or speech encoding at the subcortical level and responses are driven purely by acoustics, then a more accurate peripheral model would lead to even more similar across-stimulus response correlations.

**Figure 8:**
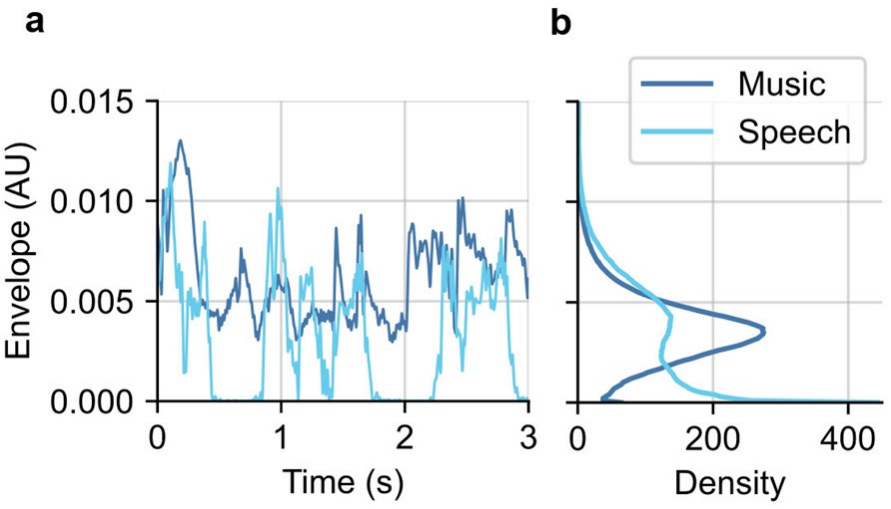
Envelope of music and speech stimuli. (**a**) Typical examples of music and speech envelopes. Each line represents an excerpt of one example stimulus from each class. (**b**) The probability density of the envelope values from all music and speech stimuli that were presented.

While the ANM regressor was better than HWR for both stimulus classes, the difference between HWR and ANM was much larger for music than for speech, with some subjects showing no HWR music response at all. We believe this discrepancy results from the nonlinearities of the auditory system interacting differently with the two stimulus classes, which have distinct acoustics^33^. We analyzed the envelope marginal distributions from this study’s stimuli and found differences (Fig. 8), corroborating previous analyses of stimulus modulations^33^. Despite designing our stimuli to have matched spectra (see Methods), the music envelope tended toward a non-zero mode (Fig. 8b), while the speech envelope had more extreme values punctuated by many silences (leading to a mode of zero). A recent pre-print tested several regressors of increasing complexity for deriving the subcortical response to speech and found that adaptation was a key component^50^. Given music’s relative lack of silences and less extreme amplitude variations, the brain’s response to music would have shown more adaptation than to speech. The ANM regressor includes adaptation, and both stimuli provided clear responses. The HWR regressor does not include adaptation, which may be why it yielded a speech response (albeit poorer than the ANM) but not a music response. After investigating subcortical responses, we further extended the analysis to cortical responses using the TRF paradigm. Compared with the traditionally used envelope as acoustical stimulus feature, the proposed ANM regressor gave improved prediction accuracy and responses to music and speech that became more similar to each other but did not converge (r = 0.33 and 0.48 for envelope and ANM, respectively). The main takeaway from this is that accounting for peripheral nonlinearities does not fully explain differences between cortical encoding of the acoustics of music and speech as it nearly does for subcortical responses (where the correlation changed from 0.00 to 0.86 when the ANM was used), suggesting that there is differing encoding or processing between music and speech that occurs predominantly at the cortical level.

There are alternatives to modeling biological nonlinearities directly, as we did here. One such paradigm involves binning the stimulus envelope by intensity, so that a set of kernels is fit that can reflect nonlinear changes in response size as well as the latency shifts that tend to come with changing stimulus level^51,52^. This approach has the advantage of not making specific assumptions about response changes with level, but there are drawbacks, too. It increases the model’s parameter count severalfold and, because it is multivariate, it can no longer be fit efficiently using Fourier division. More importantly, because it is envelope based, the method would also need to be adapted to produce subcortical responses, which would be non-trivial.

While the subcortical music and speech responses we measured here were very similar, it does not rule out specialized subcortical processing based on stimulus class. If such processing took place in a brain region where the fibers were not commonly oriented then it would be invisible to EEG^53^. It could also be that the task listeners were engaged in here—counting epochs of music and speech within a block—did not engage the neural mechanisms we set out to investigate. It is possible that a task that involved more critical listening, while still being the same task across conditions to avoid confounding results, could reveal effects missed by the current study.

Taking a step back from the main question, it is worth considering the improvement in subcortical responses offered by the newly developed ANM regressor over the previously used HWR. When using the HWR, the response to the speech stimuli was generally present, but included only wave V and lacked the earlier waves I and III, consistent with prior studies^32,54^. The response to music, however, as shown in Fig. 2, was noisy and failed to even show a distinct wave V. In contrast, the ANM-derived ABRs to both speech and music showed robust canonical waveforms with wave V in all subjects accompanied by waves I and III in many, each reflecting activity from distinct early neural generators (Fig. 3). Considering the improved morphology and the better model prediction, we recommend the ANM regressor or similar for future studies of natural sound encoding unless there is a scientific reason otherwise, as simpler models (for example, lacking adaptation) have been shown to be less predictive^50^. Another advantage of the ANM regressor is that the computational model that we used is able to simulate the auditory nerve responses in ears with hearing loss by adjusting frequency-specific parameters related to the outer hair cell and inner hair cell damage^35,36^. Different types of hearing loss will affect the ABR in different ways^55^, so comparing predictions of ANM regressors with different parameters for an individual subject may prove informative.

While cortical responses did not become identical when replacing the envelope regressor with the ANM regressor, they did become more similar. The remaining difference leads to a similar question for cortical responses that was addressed in this study for subcortical responses: is the difference due to a regressor that does not adequately account for nonlinearities, or is it due to true higher-order processing differences which are not captured by the acoustical regressor? The answer for the cortex is likely both, though the relative contributions and the specific higher order phenomena that impact the cortical responses are subjects in need of further study.

In studies using TRF methods to investigate speech-specific features such as lexical or semantic information^47,56,57^, it is a standard practice to include low-level acoustical features in the model so that their contribution can be regressed out. A simple envelope regressor can be used, but complex stimulus acoustical representations such as the spectrogram-based envelope^44^ and amplitude binned envelopes^51^ can be more effective. Based on the increased similarity between cortical responses observed in the present study, the ANM regressor is also likely to be an improvement over the simple envelope in filling this role because it more accurately models peripheral encoding. A recent study adding modeled inferior colliculus responses to TRF computation corroborates this idea^58^. In fact, future studies could take the approach of using regressors that model the auditory system up to (but not including) the level that is the focus of investigation.

## Methods

### Participants

All subjects gave informed consent prior to the experiment and were compensated for their time. Data collection was conducted under a protocol approved by the University of Rochester Subjects Review Board (#66988). All methods were performed in accordance with the relevant guidelines and regulations.

There were 24 subjects who participated in this experiment. All subjects had audiometric thresholds of 20 dB HL or better from 500 to 8000 Hz, self-reported normal or correctable to normal vision, and indicated English as their primary language.

Two subjects were excluded: one self-withdrew partway through the experiment, and technical problems during data collection led to unusable data for another subject. Therefore, after excluding the two subjects, there were 22 subjects (11 male and 11 female) with an age of 22.7 ± 5.1 (mean ± SD) years that we included in the analysis.

### Stimulus presentation

Subjects were seated in a sound-isolating booth on a chair in front of a 24-inch BenQ monitor with a viewing distance of approximately 60 cm. Stimuli were presented at an average level of 65 dB SPL and a sampling rate of 48,000 Hz through ER-2 insert earphones (Etymotic Research, Elk Grove, IL) plugged into an RME Babyface Pro digital sound card (RME, Haimhausen, Germany). The stimulus presentation for the experiment was controlled by a python script (Python Programming Language) using a custom package, *expyfun*^59^.

### Stimulus types

#### Music & speech dataset

A broad sample of music and speech stimuli was ensured by selecting clips from many genres and contexts. Six genres of music (acoustic, classical, jazz, hip-hop, metal, and pop) without vocals and six types of speech (English audiobook, Chinese audiobook, interview, instructional lecture, news, and presentation) were the main stimuli analyzed in the study. The English audiobooks were purchased and were the same as used in prior studies^30,32^, whereas the Chinese audiobooks were recorded by lab members. Other music and speech stimuli were collected from diverse sources that were Creative Commons (CC) licensed (See Supplementary Tables S1 & S2 for details).

#### Stimulus processing

First, all the stereo stimuli were converted to mono by averaging the two channels. Some of the stimuli were originally sampled at 44.1 kHz, so these were resampled to 48 kHz. Then, we normalized the volume of the music stimuli and removed silence from the speech stimuli.

In order to control for any large amplitude changes in the music stimuli, they were divided by their slow envelope (low pass filtered at 0.1 Hz) to make the overall amplitude flatter. This did not affect local dynamics and instead functioned like “turning the volume up” during quieter parts of a piece. The flattened signal was created as

$$x_{normalized} = x/\left( {e + 0.1\sigma_{e} } \right),$$

where ${x}_{normalized}$ is the flattened signal, $x$ the stimulus waveform, $e$ the envelope of the waveform low-passed at 0.1 Hz, and ${\sigma }_{e}$ the standard deviation of the envelope. The $0.1{\sigma }_{e}$ term prevents division by numbers close to zero.

The speech stimuli were processed by automatically cutting out any silent period that was longer than 0.5 s using tools previously developed by our lab^30,32^. We also manually cut out laughter and applause from the audience.

Finally, both music and speech were spectrally matched to the average spectrum over all stimuli. We separated the stimuli into 28 bands from 50 to 22,050 Hz with a spacing of 1/3 octaves using a 6^th^ order Butterworth filter. Then, the mean powers for each band were computed across all trials and were used to match the powers of every stimulus:

$$x_{n} = \mathop \sum \limits_{k = 1}^{K} \tilde{x}_{n, k} \sqrt {\overline{P}_{k} /P_{n,k} } ,$$

where ${x}_{n}$ is the spectrally matched $n$-th trial of the stimulus, $k$ the $k$-th frequency band, $K$ the total number of frequency bands ($K=28$, in our case), $n$ the $n$-th trial of the stimulus, ${\widetilde{x}}_{n, k}$ the $n$-th trial stimulus at the $k$-th frequency band,${P}_{n,k}$ the power of ${\widetilde{x}}_{n, k}$, and ${\overline{P}}_{k}$ the mean power of $k$-th frequency band averaged across trials.

### Trial presentation and task

Twelve types (six genres of music and six types of speech) of 12 s stimuli clips were presented. There were 40 trials for each type with shuffled order. Between trials, there was a 0.5 s pause.

To collect cortical activity at the same time, subjects needed to be kept alert during the experiment. Therefore, subjects were asked to do a mathematical comparison task while listening to the stimuli. One block constituted 5, 6, or 7 trials, containing a random number of music and speech trials. At the end of each block, subjects were asked to report whether there had been more music trials or speech trials within that block by clicking a button with a mouse. If a subject did not respond at the end of a block, the experiment paused. This task was not related to any EEG analysis we did, but ensured that subjects maintained some level of alertness and did not fall asleep for extended periods during the session.

### EEG data acquisition

The EEG signal was recorded using BrainVision’s PyCorder software (Brain Products GmbH, Gilching, Germany; RRID:SCR_019286). We collected both subcortical and cortical signals at the same time. For subcortical (ABR) activity, Ag/AgCl electrodes were placed frontocentrally (FCz, active non-inverting), left and right earlobes (A1, A2, inverting references), and the frontal pole (Fpz, ground). For cortical activity, 32-channels arranged according to the International 10–20 system were used. The average of the two electrodes TP9 and TP10 was used as the reference.

The ABR electrodes were plugged into an EP-Preamp system (Brain Products GmbH, Gilching, Germany) which was connected to an ActiCHamp (Brain Products GmbH, Gilching, Germany) recording system. The 32-channel active electrode system was plugged directly into the ActiCHamp. We verified that all the electrode impedances were below a threshold of 5 kΩ before the experiment started. Both cortical and subcortical signals were recorded at a sampling frequency of 10 kHz.

### Subcortical response derivation

#### EEG preprocessing

Clock drift can lead to timing differences between the EEG system and the sound card. To determine the actual sampling frequency of the EEG recording, we made a drift trigger stamped at 20 ms before the stimulus ended. The actual sampling frequency was then calculated by dividing the number of samples between the start trigger and the drift trigger by the offset of the two triggers (12 s stimulus duration − 0.02 s = 11.98 s)^30^, and the EEG was resampled so that it corresponded exactly with the stimulus presentation.

The subcortical EEG data were high-passed at 1 Hz using a first-order causal Butterworth filter to remove slow drift in the signal. We also used a second-order infinite impulse response notch filter to remove 60 Hz noise and its multiples (120 Hz and 300 Hz specifically) with a width of 5 Hz. We then averaged the left and right channels as the final subcortical EEG signal.

#### Deconvolution model for ABR

As described in Maddox and Lee^32^, we defined an encoding model of the ABR as shown in Fig. 1a. The stimulus with a non-linearity applied (i.e., regressor) was the input $x$, the EEG signal was the output $y$, and the ABR was the impulse response of a linear system.

The computation was performed in the frequency domain,

$$response = {\mathcal{F}}^{ - 1} \left\{ {\frac{{\mathop \sum \nolimits_{n} b_{n} X_{n}^{*} Y_{n} }}{{\frac{1}{N}\mathop \sum \nolimits_{n} X_{n}^{*} X_{n} }}} \right\},$$

where *response* denotes the derived impulse response (ABR), $X$ the FFT of the stimulus with the non-linearity applied (i.e., regressor), $Y$ the FFT of EEG signal, * the complex conjugate, ${\mathcal{F}}^{-1}$ the inverse FFT, ${b}_{n}$ the averaging weight of the $n$-th trial (see below), $N$ the total number of trials, and $n$ the trial index.

When computing the response, we followed a Bayesian-like process^60^ to account for variations in noise level, so that noisier trials were weighted less in the average. The EEG recording from each trial was weighted by its inverse variance, $1/\sigma_{n}^{2}$, relative to the sum of the inverse variances of all trials^30^:

$$b_{n} = \frac{{1/\sigma_{n}^{2} }}{{\mathop \sum \nolimits_{n} 1/\sigma_{n}^{2} }}.$$

#### Regressors

We analyzed the response using two different regressors:

1. Half-wave rectified stimulus (HWR; Fig. 1b, upper).
  The half-wave rectified stimulus regressor was created by first taking the positive values of the stimulus waveform and downsampling it to 10 kHz. This positive regressor was then used as the input to the encoding model shown in Fig. 1a (i.e., $x$). Then, a second calculation was done, taking the negative values, inverting their sign, and downsampling as before. We ran the deconvolution two times separately with the positive and negative regressor. The final ABR response for each specific epoch of each subject is the average of the positive and negative responses^32^.
2. Auditory Nerve Modeled Firing Rate (ANM) from the Auditory periphery Model (Fig. 1b, lower).
  We used a computational auditory periphery model created by Zilany et al.^35,36^ and its adapted python package version^61^ to generate simulated auditory neural responses. This computational model is a phenomenological model for the early auditory pathway, which can transform the stimulus waveform to human auditory representation of that acoustical signal. The model encompasses detailed neural encoding of the inner hair cells (IHC), outer hair cells (OHC) as well as the auditory nerve. Thus, it was used in our study to account for the peripheral nonlinearity effects.
  We used the auditory nerve firing rate in our analysis to investigate if the non-linear effect in the auditory pathway could compensate for the acoustical differences of music and speech. Stimuli were upsampled to 100 kHz as required by the model and converted to a pressure waveform with units of pascals at 65 dB SPL. We specified the characteristic frequency (CF) from 125 Hz to 16 kHz with intervals of 1/6 octaves. The firing rate was summed over all the CFs and downsampled to be used as the final regressor, denoted as ANM.

When deriving the ABR, we generated the ANM regressors twice. For the first time, the unaltered stimuli were input to the computational model. For the second time, we inverted the polarity of the stimuli before they were input into the model. The two polarities were used to derive two responses that were averaged to get the final ABR. This processing method with inverted polarity mostly cancelled out the stimulus artifact. We measured the lag between the stimulus and the model generated regressor from the cross-correlation between click waveform and the ANM regressor and found a 2.75 ms lag. This lag in the regressor would have the effect of shifting the derived responses to the left. Thus, when calculating the ABRs, we compensated for this lag by shifting the deconvolved responses 2.75 ms to the right.

The averaged ABR waveforms for each type of stimulus were computed across the 22 subjects. Then, the general music-evoked ABR was computed by averaging all the responses of the six genres of music. The general speech-evoked ABR was computed using the same method with the six types of speech responses. From the deconvolution computation, we were able to get the response within the time interval of [0, 12] s. But due to the circular nature of discrete frequency domain deconvolution, the response can be represented as [− 6, 6] s by concatenating the last 6 s and the beginning 6 s of the response. For display and analysis of the response waveform, we limit the time interval to [− 200, 600] ms.

### Statistical analysis for subcortical responses

#### Response signal-to-noise ratio (SNR)

To evaluate the quality of the responses, we estimated the SNRs of each ABR waveform using the equation

$$SNR = 10\log_{10} \left[ {\frac{{\sigma_{S + N}^{2} - \sigma_{N}^{2} }}{{\sigma_{N}^{2} }}} \right],$$

where ${\sigma }_{S+N}^{2}$ is the variance of the ABR waveform in the time range of [0, 15] ms, and ${\sigma }_{N}^{2}$ is the variance of the noise calculated by averaging the variance of every 15 ms segment in the pre-stimulus baseline, covering a time range of [− 200, − 20] ms. The difference of ${\sigma }_{S+N}^{2}$ and ${\sigma }_{N}^{2}$ in the numerator provides an estimate of the signal variance ${\sigma }_{S}^{2}$. If ${\sigma }_{N}^{2}$ is too high and greater than ${\sigma }_{S+N}^{2}$, ${\sigma }_{S}^{2}$ becomes negative (an impossibility), indicating that there is not enough signal for the SNR to be estimated.

#### Correlation between the predicted and the real EEG

To compare the power of the regressors to predict EEG, we computed the correlation coefficient between the predicted EEG from the responses derived using the two regressors and the real EEG data. To get the predicted EEG, we used the general stimulus category kernels which are the general music- and general speech-evoked ABR derived from the two regressors of the encoding model ([0, 200] ms time range), and convolved the kernels with the corresponding types of stimuli regressors (e.g., the general music ABR convolves with the regressors of all music stimuli). The Pearson correlation coefficient between the predicted and real EEG was computed, and then was compared using paired t-test between the two regressors.

#### Spectral coherence analysis

We also used a spectral coherence analysis to compare the power of the regressors in predicting EEG on a per-frequency basis. This method served as a normalized correlation between the predicted EEG and the real EEG data but in different frequency bins. All of the predicted and the real EEG data were sliced into segments based on pre-determined window sizes (0.2 s in our study), which then determined the frequency bins. The coherence of each frequency bin was calculated as

$$C_{xy} \left( f \right) = \frac{{E\left[ {X_{i}^{*} \left( f \right) Y_{i} \left( f \right)} \right]}}{{\sqrt {E\left[ {X_{i}^{*} \left( f \right) X_{i} \left( f \right)} \right] E\left[ {Y_{i}^{*} \left( f \right)Y_{i} \left( f \right)} \right]} }},$$

where ${C}_{xy}\left(f\right)$ denotes the coherence between signal $x$ and $y$ at frequency bin $f$, $E$[ ] the expected value across slices, * the complex conjugate, ${X}_{i}$ the FFT for predicted EEG slice $i$, and ${Y}_{i}$ the FFT for real EEG data slice $i$.

We then randomized the order of the predicted and real EEG and computed the spectral coherence of mismatched trials. The median of the mismatched coherence was used as the noise floor.

#### Wave V latencies

To verify the waveform agreement, two authors (TS and RKM) manually picked the wave V peak latencies of the click-evoked ABR, the general music- and general speech-evoked ABR (from the ANM regressor) from each of the 22 subjects. A paired t-test was used to compare the latency differences between general music- and general speech-evoked ABRs.

#### Correlation between music- and speech-evoked ABR waveforms

The Pearson correlation coefficients of the music- and speech-evoked ABR derived from the two regressors were computed for the waveforms in the time range of [0, 15] ms. The Wilcoxon signed-rank test was then used to compare the similarity between music- and speech-evoked ABR waveforms.

### Cortical response derivation

#### EEG preprocessing

The clock drift was corrected as described above.

For cortical responses derived through deconvolution, the preprocessing was the same as the subcortical EEG. The only difference is that the EEG signal was high-passed at 0.1 Hz. For the TRF analysis, preprocessing was done as instructed by Crosse et al.^40^. The signal was high-passed at 0.5 Hz and low-passed at 30 Hz with bidirectional zero-phase FIR filter. The EEG data was then downsampled to 125 Hz. The first 1 s of each epoch was removed to avoid onset effect.

#### TRF model for cortical responses

We employed the temporal response function (TRF)^62,63^ to investigate the cortical neural responses using mTRF toolbox^40^ and its adapted *python* version. The TRF paradigm uses time-resolved regularized linear model (ridge regression) to find how the changes of specified stimulus features are linearly reflected in brain activity^37,40^. The time lag window of [− 50, 400] ms was used to fit the TRF model. The prediction accuracy was assessed in a cross-validation with 10 folds.

#### Regressors

We included the amplitude envelope as a comparison to the proposed ANM regressor. The broadband amplitude envelope was extracted from the Hilbert transform of the stimulus waveforms. We opted to use this regressor rather than HWR so that our results would be comparable to what other labs have published. The ANM regressor was extracted as described above. Both regressors were then downsampled to 125 Hz with anti-aliasing filtering to match the EEG sampling frequency.

### Statistical analysis for TRF

#### Correlation between the predicted and the real EEG

The estimation of prediction accuracy of TRF model was obtained through the cross-validation process. A paired t-test was used to compare the accuracy between the two regressors within each stimulus category.

#### Correlation coefficient between music and speech TRFs

The Pearson correlation coefficients of the music and speech TRF derived from the two regressors were computed for the TRF weights in the time range of [0, 360] ms. We performed a linear mixed effects model with the regressor as the fixed effect, and the subject and EEG channel as the random effects using *lme4*^64^ and *lmerTest*^65^ in *R*^66^:

$$Coefficient\sim regressor + (1 | subject) + (1| channel)$$

The *mne-python* package^67^ was used to show the responses for each channel, the topographies of pivotal time points, and the global field power (GFP).

### Statistical analysis for stimulus acoustics

To investigate the intrinsic acoustical differences of music and speech, we analyzed the envelope statistics of the stimuli. We took the half-wave rectified stimulus and applied a low pass filter with a cutoff at 40 Hz to extract the stimulus envelope. The marginal probability density function was used to show the distribution of the envelope for music and speech.

### Supplementary Information

Supplementary Information.

## Data Availability

The EEG recording data are available in EEG-BIDS format at OpenNeuro.org (10.18112/openneuro.ds004356.v1.0.0). The Python code for this study is available on GitHub (https://github.com/maddoxlab/Music_vs_Speech_abr).
