# Supplementary material for: Subcortical responses to music and speech are alike while cortical responses diverge
Source: Sci Rep. 2024 Jan 8;14:789. doi: 10.1038/s41598-023-50438-0 (PMC10774448; doi:10.1038/s41598-023-50438-0)
Supplement: Supplementary file 1 — Supplementary Information. [file 41598_2023_50438_MOESM1_ESM.docx]

**Subcortical responses to music and speech are alike while cortical responses diverge**

Tong Shan^1, 2, 3^, Madeline S. Cappelloni^1, 2, 3^, Ross K. Maddox^1, 2, 3, 4^*

^1^ Department of Biomedical Engineering, University of Rochester, United States

^2^ Del Monte Institute for Neuroscience, University of Rochester, United States

^3^ Center for Visual Science, University of Rochester, United States

^4^ Department of Neuroscience, University of Rochester, United States

Corresponding Author:

Ross K. Maddox, Department of Biomedical Engineering & Department of Neuroscience, University of Rochester, USA. Email: [ross.maddox@rochester.edu](mailto:ross.maddox@rochester.edu)

# Supplemental Materials


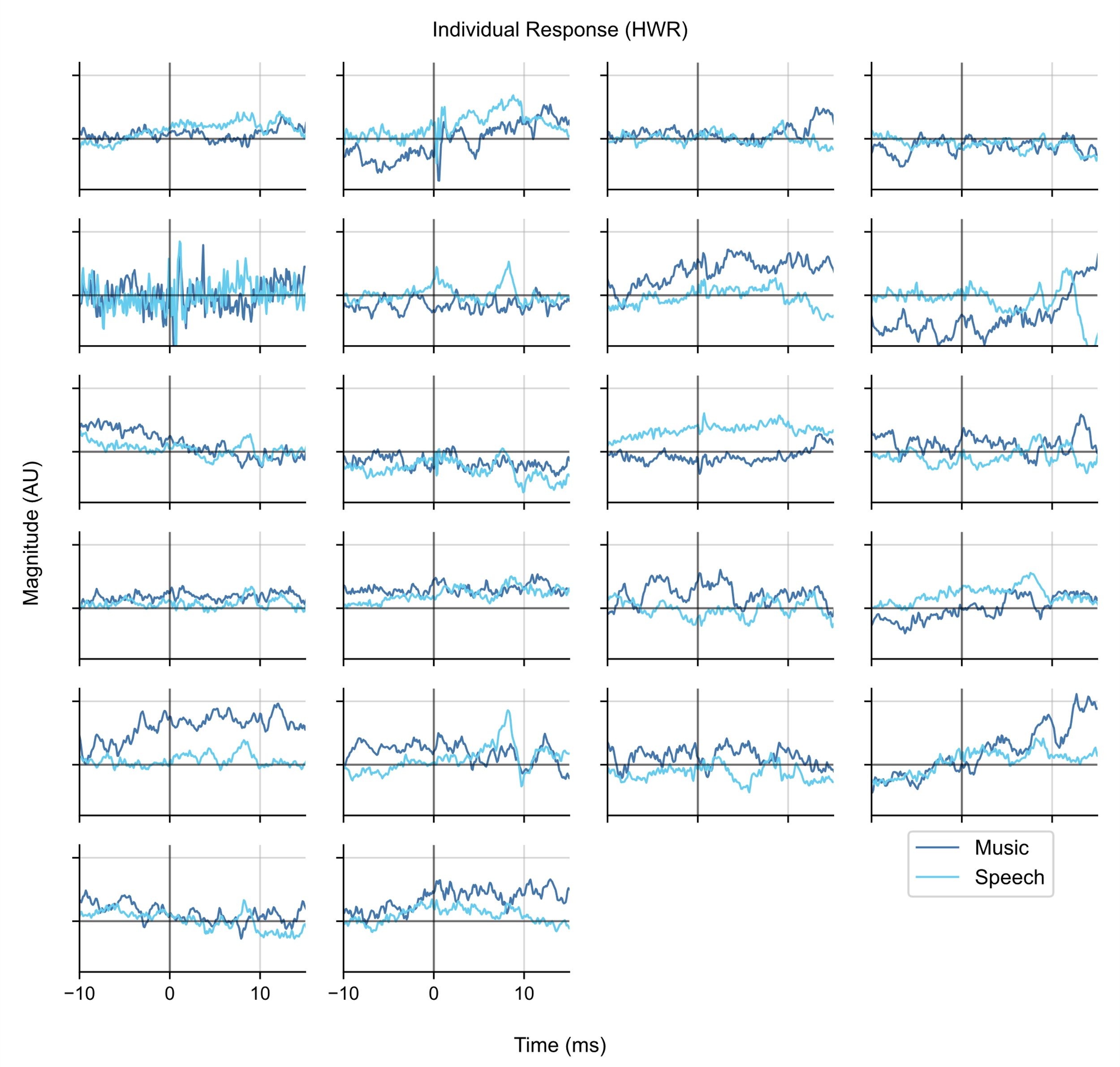


**Figure S1. Responses of all subjects using the HWR regressor.** Only a few subjects showed clear wave V for speech responses, but not for music.


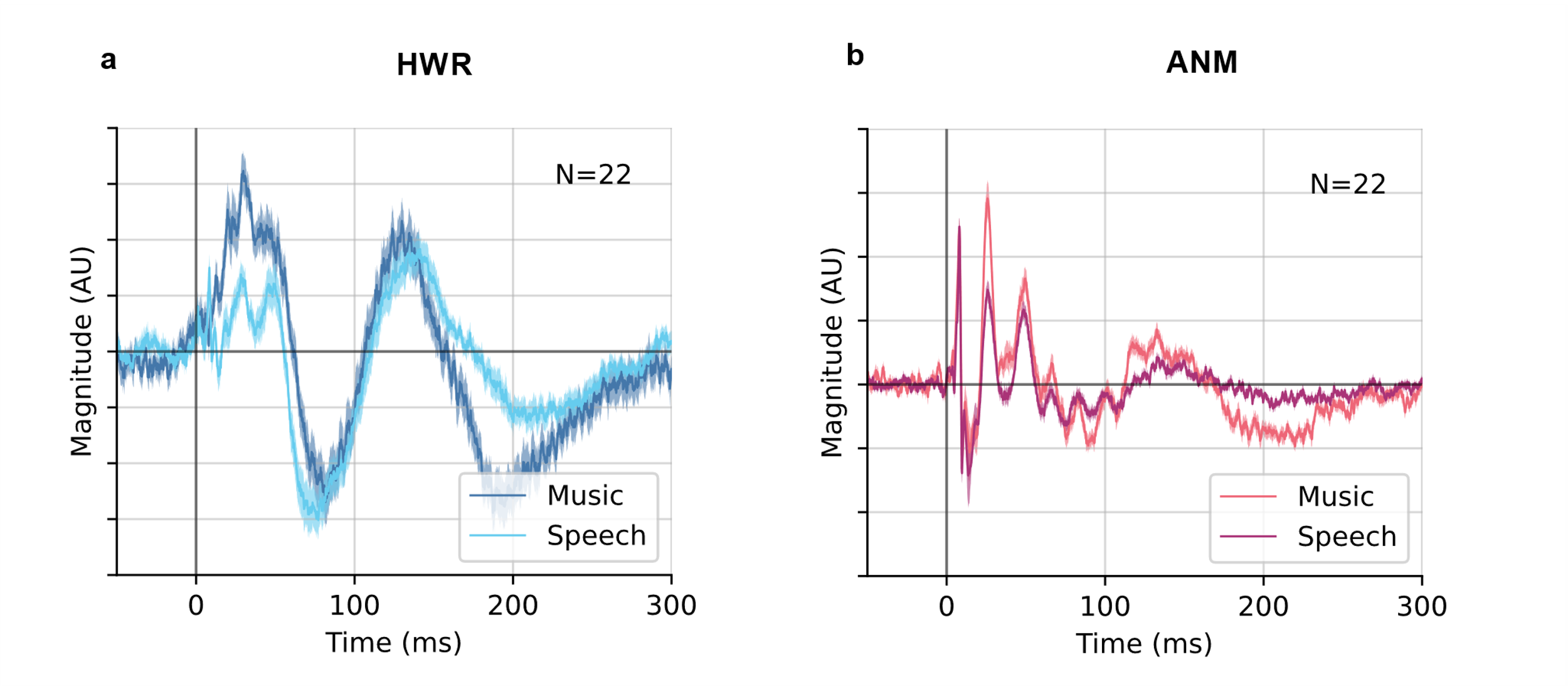


**Figure S2. Extended grand averaged response using HWR and ANM regressors from –50 to 300 ms.**


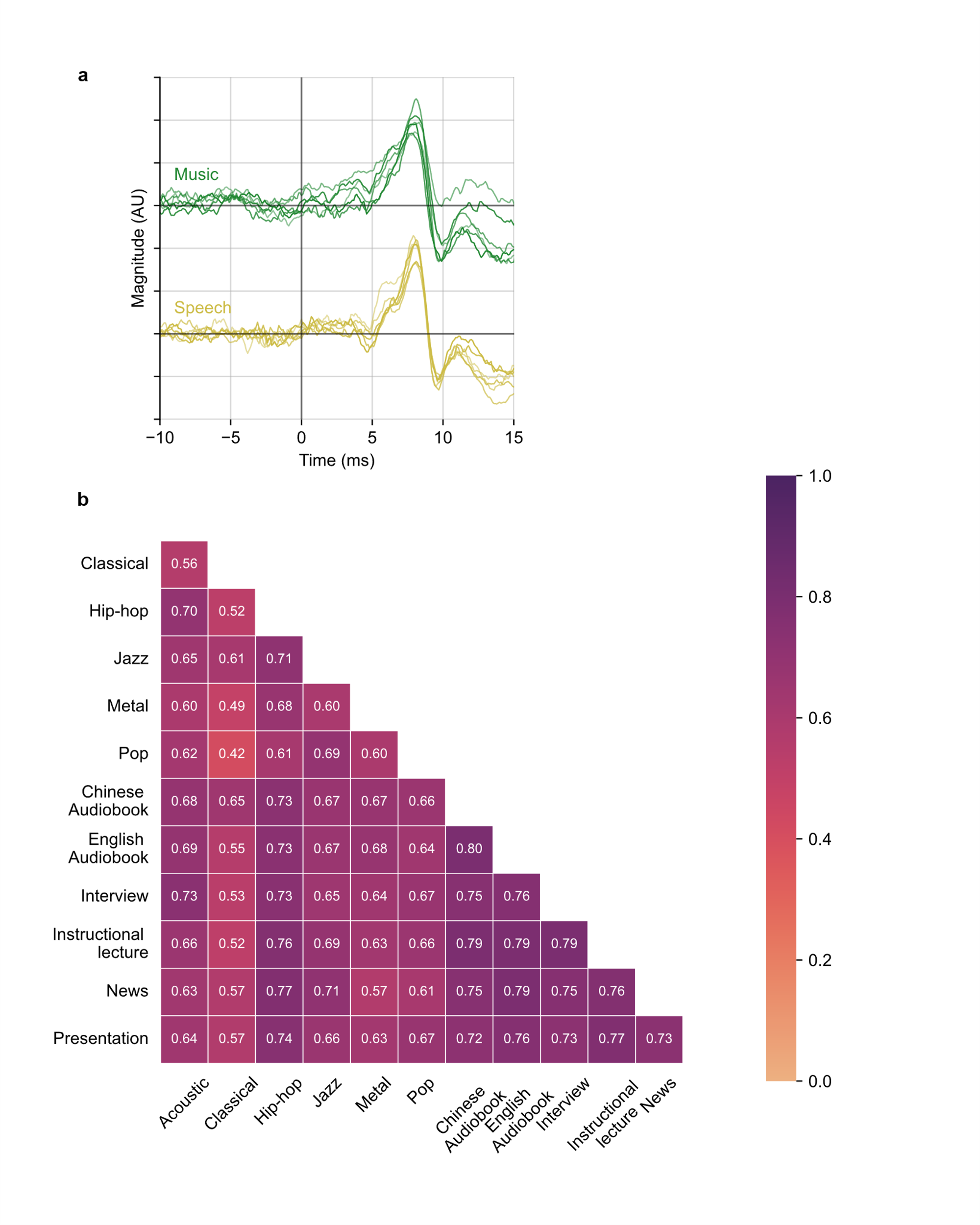


**Figure S3. a. The grand averaged ABR response for each genre of music and each type of speech stimuli.** The upper lines are music-evoked ABRs. The lower lines are the speech-evoked ABRs. The different shades of lines represent different genres of music or different types of speech. No systematic differences are observable across genres for either music or speech, while the variance among music genres is larger. **b.** **Pair-wise correlation coefficient (Pearson’s r) of ABR responses for each genre of music and each type of speech stimuli.** Numbers shown in the matrix are the median across subjects. The coefficients of speech types are higher than that of music genres within their categories (*p* = 8.99 × 10^-50^; one-tailed Wilcoxon signed-rank test).

**
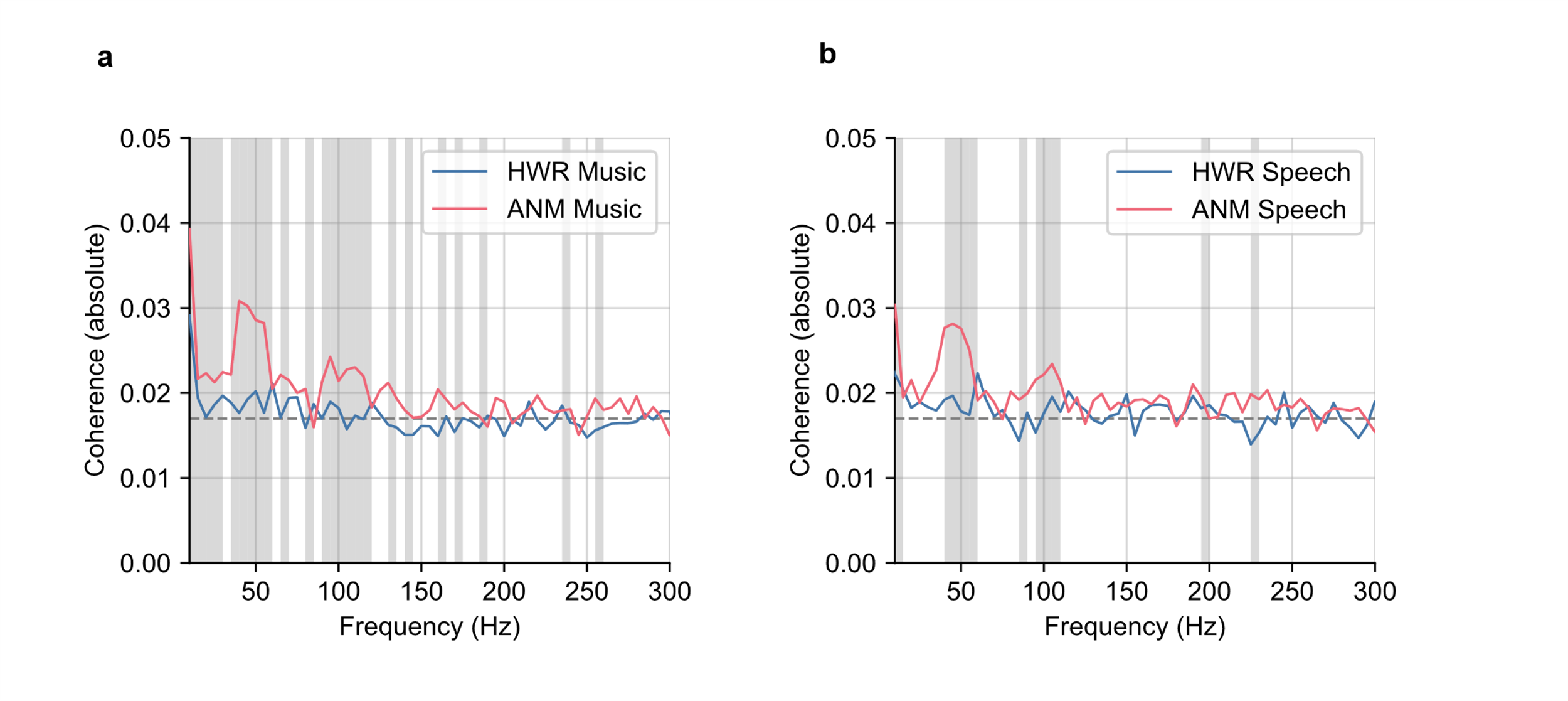
**

**Figure S4. Spectral coherence with subcortical kernel. a. Spectral coherence for music trials with subcortical kernel. b. Spectral coherence for speech trials with subcortical kernel.** The solid lines are the median absolute value of spectral coherence for each regressor in each frequency bin. The shaded areas show frequency range where the coherence for ANM regressor was significantly higher than the HWR regressor (*p* < 0.05; Wilcoxon signed-rank test, FDR corrected). The dashed lines are noise floor.

**
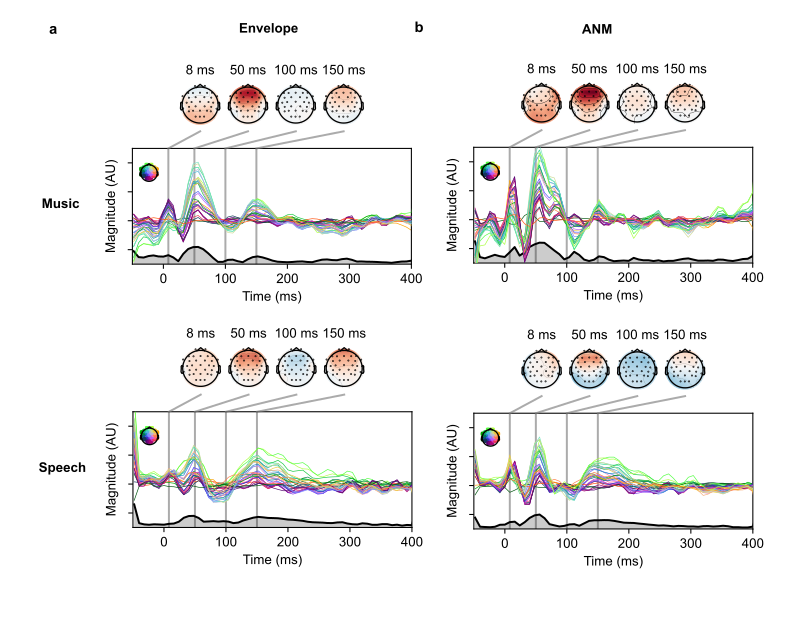
**

**Figure S5. Grand average TRFs weights for 32 channels. a. TRFs derived from envelope regressor. b. TRFs derived from ANM regressor.** Topographies are selected from the time points of pivotal peaks (same as **Figure 6)**. The thick black line with grey under-line area represents the global field power (GFP) of the derived response.

**
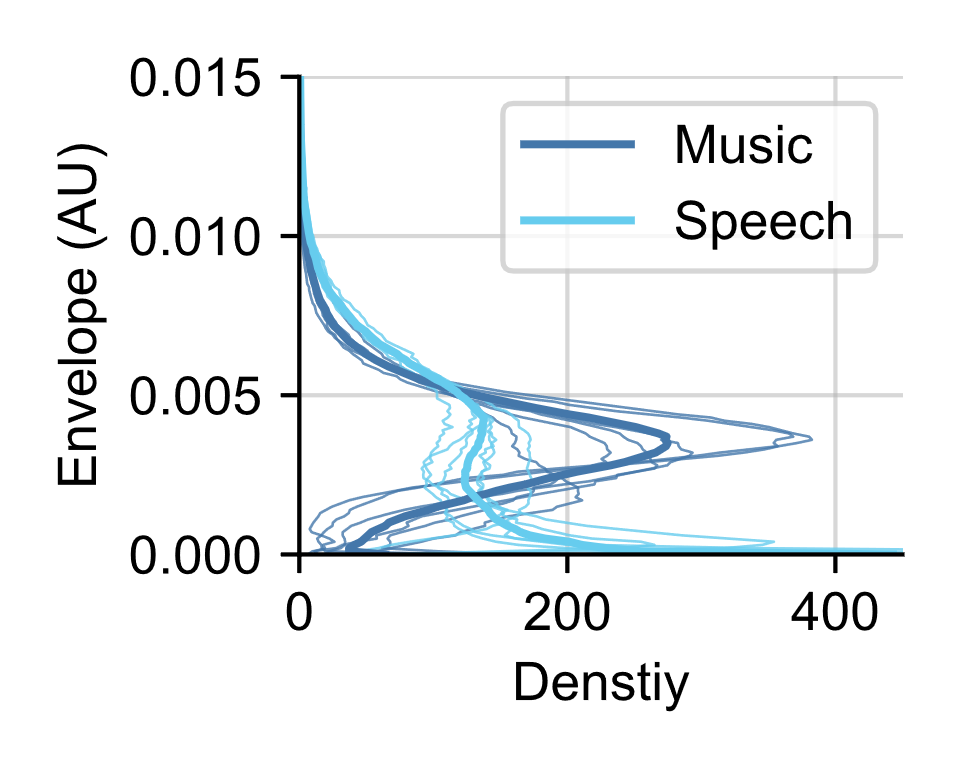
**

**Figure S6. Envelope probability density function of music and speech.** Thinner darker blue lines represent each genre of music and thinner lighter blue each type of speech. Thick darker blue line is the pooled music of each genre and thick lighter blue line is pooled speech of each type.

**Table S1. Music Stimuli.**

| Type | Sources | Link | Content |
| --- | --- | --- | --- |
| Classical | Free Music Archive | https://freemusicarchive.org/ | Borromeo - String Quartet – 03 – Mozart String Quartet No16 in E-flat Major  MIT Symphony Orchestra – 01 – The Merry Wives of Windsor Overture Nicolai 004  Scott Holmes Music-Conclusion-FMA 000 |
| Jazz |  |  | Till Paradiso-Be Part of My Universe-FMA |
|  | DSD100 ^57^ | https://sigsep.github.io/datasets/dsd100.html | 030 - Patrick Talbot - A Reason To Leave  081 - Patrick Talbot - Set Me Free |
| Acoustic |  |  | 021 - James May - On The Line  044 - Tom McKenzie – Directions  062 - Cristina Vane - So Easy |
| Hip-hop |  |  | 001 - ANiMAL - Clinic A  027 - M.E.R.C. Music – Knockout  052 - ANiMAL - Easy Tiger |
| Metal |  |  | 018 - Hollow Ground - Ill Fate  043 - Timboz – Pony  069 - Hollow Ground - Left Blind  098 - Wall Of Death - Femme |
| Pop |  |  | 024 - Leaf - Come Around  037 - Speak Softly - Broken Man  041 - The Mountaineering Club - Mallory |

**Table S2. Speech Stimuli.**

| Type | Sources | Link | Content |
| --- | --- | --- | --- |
| Chinese Audiobook | Recorded | N/A | *My Old Home* by Lu Xun  *Kong Yiji* by Lu Xun |
| English Audiobook | Polonenko and Maddox (2021) ^30^ | https://datadryad.org/stash/dataset/doi:10.5061/dryad.12jm63xwd | The Alchemyst (Scott, 2007)  A Wrinkle in Time (L’Engle, 2012) |
| Interview | YouTube | https://www.youtube.com/watch?v=J0WbRmlSXBU | CBC Sports - NBA Commissioner Adam Silver weighs in on the Raptors, racism, and politics \| CBC News: The National |
| Instructional lecture | Khan Academy | <https://www.khanacademy.org/science/electrical-engineering/ee-signals/ee-fourier-series/v/ee-fourier-series-intro> | Khan Academy - Fourier Series introduction (video) |
|  | YouTube | https://www.youtube.com/watch?v=9yETqNk0eMc | YaleCourses - The Deuteronomistic History: Prophets and Kings |
| News | YouTube | https://www.youtube.com/watch?v=Fy-mG4ktdS8 | BBC News - Snow Storm Report 28/2/18 |
| Presentation | TED Talk | https://www.ted.com/talks/genevieve_bell_6_big_ethical_questions_about_the_future_of_ai  https://www.ted.com/talks/nick_bostrom_what_happens_when_our_computers_get_smarter_than_we_are | Genevieve Bell - 6 big ethical questions about the future of AI  Nick Bostrom - What happens when our computers get smarter than we are? |
